# Are we willing to share what we believe is true? Factors influencing susceptibility to fake news

**DOI:** 10.3389/fpsyt.2023.1165103

**Published:** 2023-08-16

**Authors:** Michal Piksa, Karolina Noworyta, Aleksander B. Gundersen, Jonas Kunst, Mikolaj Morzy, Jan Piasecki, Rafal Rygula

**Affiliations:** ^1^Department of Pharmacology, Affective Cognitive Neuroscience Laboratory, Maj Institute of Pharmacology Polish Academy of Sciences, Krakow, Poland; ^2^Department of Psychology, University of Oslo, Oslo, Norway; ^3^Faculty of Computing and Telecommunications, Poznan University of Technology, Poznan, Poland; ^4^Faculty of Health Sciences, Department of Philosophy and Bioethics, Jagiellonian University Medical College, Krakow, Poland

**Keywords:** misinformation, fake news, susceptibility, cognitive utility, truthfulness, COVID-19, social media

## Abstract

**Background:**

The contemporary media landscape is saturated with the ubiquitous presence of misinformation. One can point to several factors that amplify the spread and dissemination of false information, such as blurring the line between expert and layman's opinions, economic incentives promoting the publication of sensational information, the zero cost of sharing false information, and many more. In this study, we investigate some of the mechanisms of fake news dissemination that have eluded scientific scrutiny: the evaluation of veracity and behavioral engagement with information in light of its factual truthfulness (either true or false), cognitive utility (either enforcing or questioning participants' beliefs), and presentation style (either sober or populistic).

**Results:**

Two main results emerge from our experiment. We find that the evaluation of veracity is mostly related to the objective truthfulness of a news item. However, the probability of engagement is more related to the congruence of the information with the participants' preconceived beliefs than to objective truthfulness or information presentation style.

**Conclusion:**

We conclude a common notion that the spread of fake news can be limited by fact-checking and educating people might not be entirely true, as people will share fake information as long as it reduces the entropy of their mental models of the world. We also find support for the Trojan Horse hypothesis of fake news dissemination.

## Introduction

As we go about our daily lives, we are constantly exposed to new information, including news reports regarding the pandemic or Russia's invasion of Ukraine, politicians' statements about domestic policy, friends' descriptions of new restaurants, and celebrity gossip. However, how do we decide what is true and what is false? This question is more pertinent today than ever. Modern social media blurs the line between facts and opinions, which opens up the opportunity for misinformation.

The ability and ease of sharing information on social media can amplify the effects of the malicious spread of fake news. Therefore, an important research challenge is to determine how people assess the veracity of the information they encounter and how those decisions affect their online behavior, e.g., by ignoring or sharing it. Understanding how people make decisions about such actions is important for many fields, ranging from politics and national security through finance to education and public health.

Apart from information's objective truthfulness, when assessing people's susceptibility to fake news, it is also crucial to consider the cognitive utility of the news, which can be defined as the ability of information to enhance or reduce people's sense of understanding the world around them (1). In 2010, Friston suggested that people strive to minimize the difference between the mental models that they use to comprehend and actual external reality to ensure that their sensory entropy remains low (2). This can be achieved either by seeking out information that strengthens the uncertain elements of the adopted mental models and/or by avoiding information that is suspected to weaken or disconfirm them. In other words, people tend to reduce the cognitive dissonance between the internal representation of reality and the actual external reality and tend to improve their sense of comprehension by actively selecting or avoiding the information on which they build their awareness (3).

There is also a range of contextual factors that may influence an individual's tendency to believe the news (4). These include presentation style elements that increase the affective load and references to a social consensus increasing the veracity of the news. Fake news is typically accompanied by a photograph that may or may not provide additional information about the content of the story, but it is often emotionally evocative and geared toward provoking shock, fear, or anger. Previous research has shown that presenting a photograph alongside a text description increases veracity ratings (5), and the emotional load increases belief in the news (6). Similarly, a reference to a source and the use of wording in the form of social consensus, e.g., “as many of us already know” or “as reported by multiple sources”, can trick people into feeling an increased sense of truthfulness (7).

Apart from problems with the evaluation of information veracity, susceptibility to fake news can also be associated with engagement with the news, e.g., a willingness to share it with peers or in social media environments, through likes, shares, comments, etc. (8). Indeed, the desire to share information within one's social circle is deeply rooted in evolution (9). As gossiping serves to build trust networks in past generations, sharing, liking, and commenting on online information in social networks reinforces trust in digital communities (10). We believe that the evolutionary trait underlying the propensity to share online information is an important index of fake news susceptibility (11).

Based on this multifactorial and multidimensional framework, we investigated how three factors, truthfulness (true vs. false), cognitive utility (congruent vs. incongruent), and presentation style (populistic vs. sober) influence the susceptibility to COVID-19 (mis)information at the level of veracity judgment and behavioral engagement with the news. The choice of the news topic was dictated by the fact that, during the data collection, most people around the globe were heavily engaged with the COVID-19 pandemic.

## Materials and methods

### Ethics statement

The study was conducted in accordance with all legal requirements regarding the conduct of scientific research in the Kingdom of Norway and the guidelines laid down in the Declaration of Helsinki. Consent was obtained from all subjects. The identical study design was approved by the Bioethics Committee of the Jagiellonian University in Krakow, Poland (1072.6120.66.2021).

### Participants

**Figure 1:**
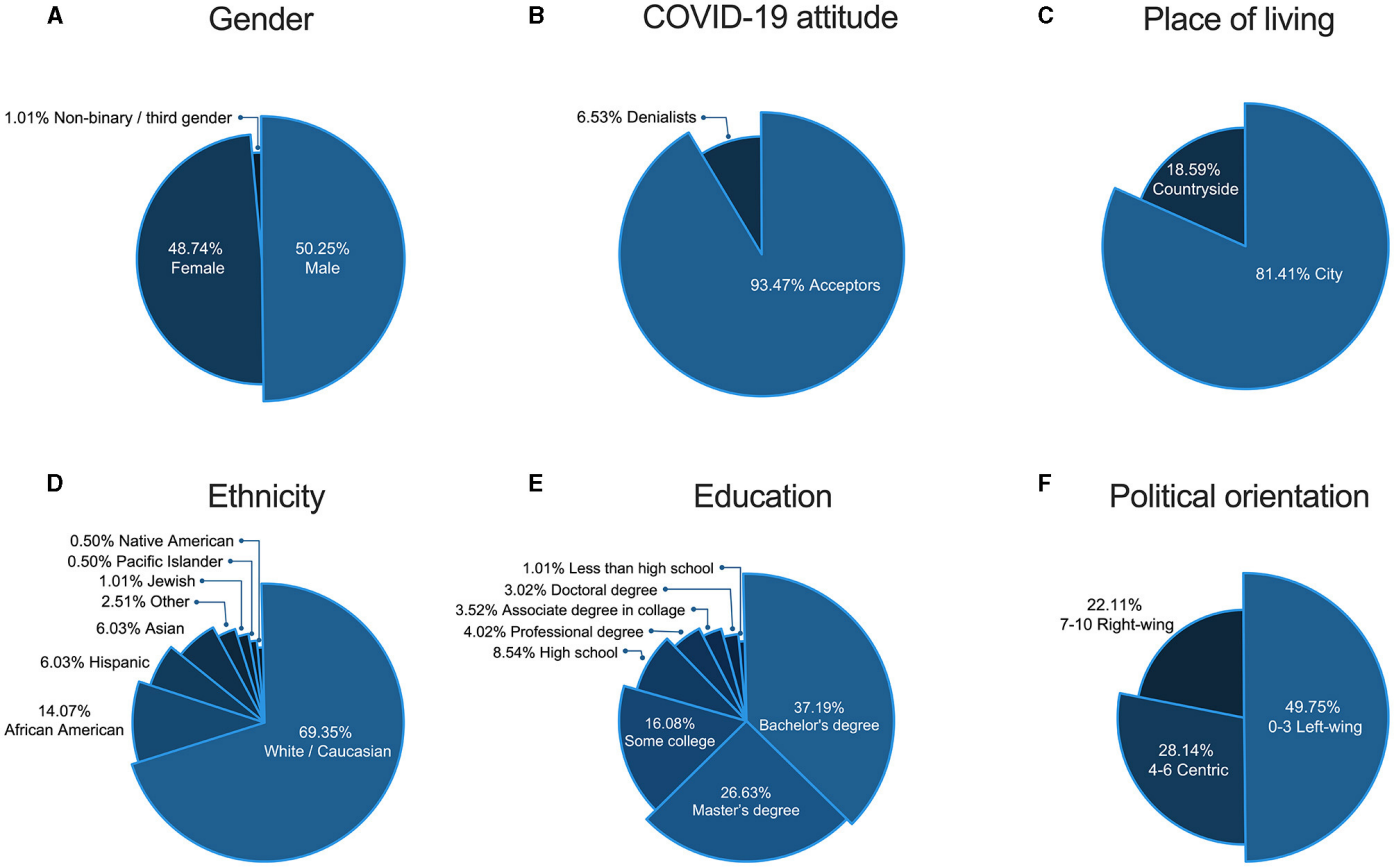
Demographic data of the researched sample include **(A)** gender; **(B)** COVID-19 attitude; **(C)** place of living; **(D)** ethnicity; **(E)** education; and **(F)** political orientation.

The sample of 201 adult Americans was recruited by Prolific Academic. To receive reliable answers, we recruited only people who had previously participated in a minimum of 100 studies and a maximum of 500 studies, with an acceptance rate of ≥95% for the submitted surveys. During the survey, the participants had to pass two attentional checks (e.g., *It is important that you pay attention to this study. Please tick “Somewhat agree*”), and all participants answered all checks correctly. Two participants who did not declare their attitude toward the COVID-19 pandemic were excluded from further analysis, as this attitude was crucial for determining the cognitive utility (congruency with one's views) of each news item (see subsection “News items”, section “Materials and methods”). The final sample (*N* = 199, M_age_= 36.32, SD = 11.11) included 186 participants who declared that the pandemic is, at least to some degree, a real threat (further called Acceptors) and 13 participants who declared that the pandemic is, at least to some degree, a hoax (further called Denialists). A summary of other demographic data is presented in Figure 1.

### News items

**Figure 2:**
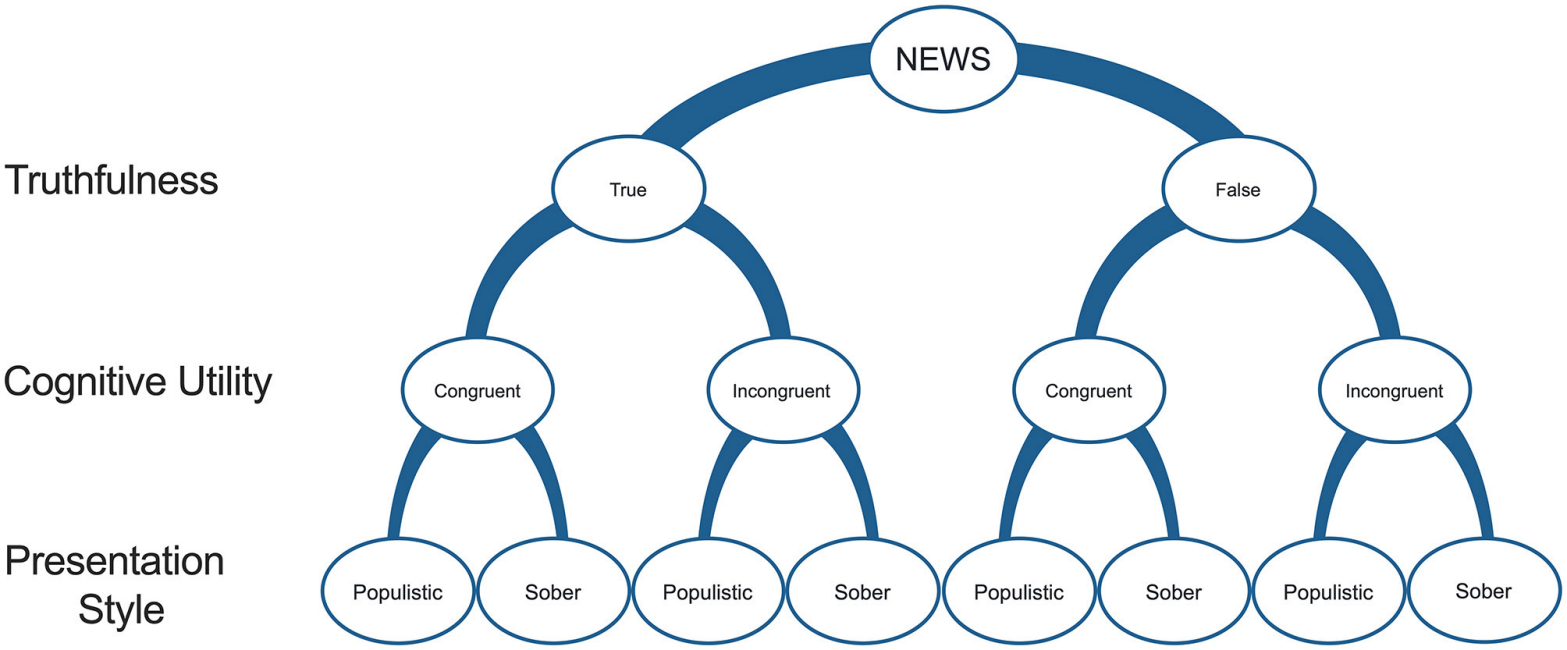
Factors (truthfulness, cognitive utility, and presentation style) of the news items.

To test which features of the news contribute the most to the susceptibility to (mis)information, we designed 80 news items that could be categorized into eight types on the basis of three factors' modalities: truthfulness (true vs. fake), cognitive utility (congruent vs. incongruent with the personal attitude toward the COVID-19 pandemic), and presentation style (populistic vs. sober; described later in this subsection). The eight possible types of items were (1) fake, congruent, and populistic; (2) fake, congruent, and sober; (3) fake, incongruent, and populistic; (4) fake, incongruent, and sober; (5) true, congruent, and populistic; (6) true, congruent, and sober; (7) true, incongruent, and populistic; and (8) true, incongruent, and sober (Figure 2). All of the news items were prepared to mimic a Facebook-like format, i.e., they consisted of a news headline, a picture, a subtext line, and the source of the information. The topic of the news was connected to the COVID-19 pandemic because most people around the globe were absorbed in it at the time of data collection, making it more ecologically valid. Half of the items presented objective truths based on information from the official WHO guidelines (12). The other half presented false information, which was invented and verified as false by the research team. Second, to investigate the effects of information utility on its valence, we designed the items to reflect the polarization in beliefs about the COVID-19 pandemic. One-half of the news was congruent with the view that the COVID-19 pandemic is real and threatening (i.e., in line with the attitude of acceptors), while the other half was created to align with the view that the pandemic is a hoax (i.e., in line with the attitude of denialists). We, thus, assumed that the news that aligns and fits with the view that the COVID-19 pandemic is real and threatening will be congruent with the attitude of acceptors (positive cognitive utility) and, at the same time, incongruent with the attitude of denialists (negative cognitive utility), and vice versa—the news claiming that COVID-19 is a hoax will be incongruent with the attitude of acceptors (negative cognitive utility) and, at the same time, congruent with the attitude of denialists (positive cognitive utility).

As mentioned above, apart from actual truthfulness and cognitive utility, a range of contextual factors (e.g., presentation style) can influence the tendency to believe and share the news (4–7). Thus, half of the news items were presented with elements that increased affective load (e.g., a sensational headline that dramatized more than the text below, accompanied by a bright, colorful, and high contrast, sensational picture), social consensus (text with references to common agreement), and references to alternative (non-mainstream) sources of information (populistic presentation). The other half was presented as sober facts, countering or lacking the abovementioned features (i.e., they had a sober presentation style). All the news items are available in an online repository (13).

The susceptibility to (mis)information was defined on two levels. The participants were asked to evaluate each item in terms of its veracity (*Do you think the news above is true?*), on a 6-point Likert scale, where 1 was *definitely false* and 6 was *definitely true*, and the probability of engagement with it (willingness to like—*On social media, I would give a “like” to this news*, and willingness to share—*I would share this news on my social media profile)*, on a 6-point Likert scale, where 1 was *totally disagree* and 6 was *totally agree*. For the internal consistency measures, please see Supplementary material.

### Statistical analysis

The data were analyzed using SPSS (version 27.0, SPSS INC., Chicago, IL, USA). Three-way repeated measures analysis of variance (ANOVA) with Sidak *post-hoc* adjustment was performed to determine the main effects of and the interactions among the factor's truthfulness (true vs. false), cognitive utility (congruent vs. incongruent), and presentation style (populistic vs. sober) on the dependent variables: veracity rating and engagement with the news.

Distribution of the data within groups was tested using the Kolmogorov–Smirnov test, and homogeneity of variances was tested using Levene's test. The sphericity of the ANOVA was verified using Mauchly's test. To determine the most influential factor out of the three investigated ones affecting veracity judgment, the scores of veracity for one modality were initially subtracted from the scores of the counter-modality in respective categories: Δ_veracity_ truthfulness = |true_veracity_ – false_veracity_|; Δ_veracity_ cognitive utility = |congruent_veracity_ – incongruent_veracity_|; and Δ_veracity_ presentation style = |populistic_veracity_ – sober_veracity_|. These differences were, then, compared using repeated measures of one-way ANOVA followed by *post-hoc* tests with Sidak adjustment. Analogous operations were performed for engagement scores.

### Procedure

The study was conducted between 14 April 2021 and 16 April 2021. Eligible participants were recruited for the study via Prolific Academic, where they found the essential information and instructions. Following informed consent, they were redirected to Qualtrics.com, where they completed the survey.

The survey consisted of 80 news items, displayed in a random order, followed by claims on a 6-point Likert scale, where 1 represented *totally disagree* and 6 represented *totally agree*:

1. *On social media, I would give a “like” to this news*.
2. *I would share this news on my social media profile*.

The news items were, then, presented again, but this time, the participants had to judge the news' veracity on a 6-point Likert scale, where 1 was *definitely false* and 6 was *definitely true*.

Participants rated the engagement and veracity in two separate series of news item presentations because prior veracity judgment might decrease their willingness to engage with the news (8).

The end of the survey consisted of a demographic questionnaire and a debrief regarding the news items. After completing the survey, the participants were compensated with 5.63 GBP.

## Results and discussion

**Figure 3:**
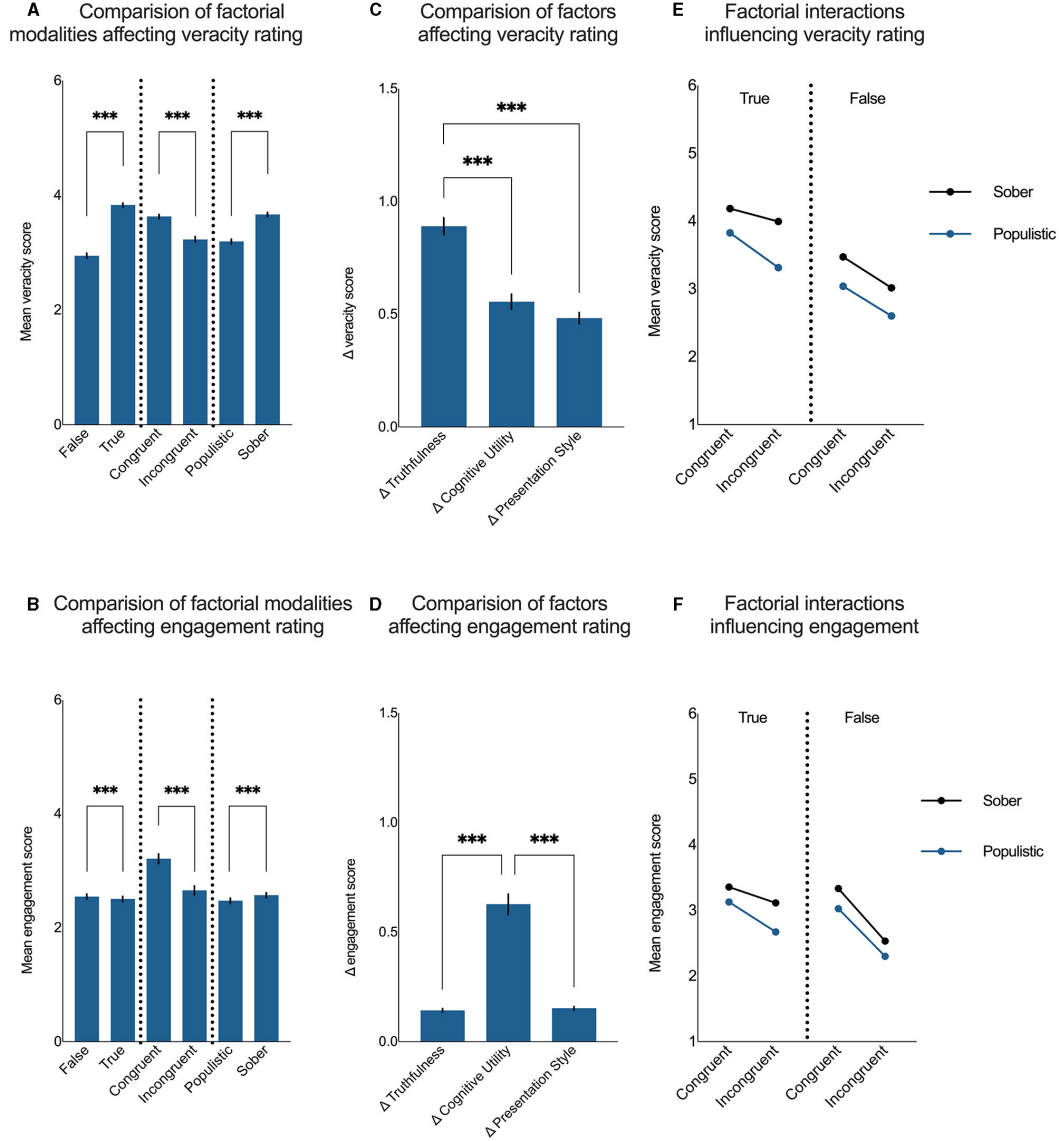
Factors and their interactions influence the susceptibility to information. **(A, B)** Demonstrate factorial modalities influencing veracity rating and engagement with the news, respectively. **(C, D)** Demonstrate the effects of truthfulness, cognitive utility, and presentation style on veracity rating and engagement with the news, respectively (Δtruthfulness = |true – false|; Δcognitive utility = |congruent – incongruent|; Δpresentation style = |populistic – sober|). **(E, F)** Demonstrate the interactions between factors affecting veracity rating and engagement with the news, respectively. The data are presented as the mean ± SEM; ***p < 0.001.

The results of our study revealed that the true news items were rated as significantly more true than those that were false [*F*_(0.81,161)_ = 408.87, *p* < 0.001, Figure 3A]. The same was observed for the news that was congruent with the rater's attitude compared with those that were incongruent [*F*_(0.82,163)_ = 84.24, *p* < 0.001, Figure 3A] and for those presented in a sober manner compared with those presented in a populistic style [*F*_(0.98,193)_ = 314.72, *p* < 0.001, Figure 3A]. Subsequent comparisons revealed that the veracity judgment was significantly (*p* < 0.001) more influenced by the actual truthfulness of the news than its cognitive utility or presentation style [*F*_(1.8,357)_ = 49.49, *p* < 0.001; Figure 3C].

While the abovementioned results seem intuitive, the behavioral engagement with the news turned out to be significantly higher for the fake news than that which was objectively true [*F*_(0.71,140)_ = 64.10, *p* < 0.001; Figure 3B]. In terms of cognitive utility and presentation style, similar to veracity ratings, the behavioral engagement with the news was significantly higher for the news items that were congruent with the participant's attitude than those that were incongruent [*F*_(0.90,178)_ = 118.30, *p* < 0.001, Figure 3B] and for those that were presented in a sober manner compared with the news items presented in a populistic manner [*F*_(0.87,172)_ = 134.05, *p* < 0.001, Figure 3B]. Engagement with the news was significantly (*p* < 0.001) more influenced by its cognitive utility than its truthfulness or the way it was presented [*F*_(1.1,216)_ = 108.45, *p* < 0.001; Figure 3D]. The observation that the actual truthfulness of the news is essential for the assessment of its veracity and not for the behavioral engagement with it suggests that, for grasping the complex nature of susceptibility to fake news, this phenomenon must be considered on at least two different levels: how people assess the veracity of given information and how likely they are to share it with their peers. These results suggest that when assessing the veracity of the news, people tend to focus mostly on its truthfulness, whereas when engaging with the news, they prefer information that is congruent with their view rather than actually true. This observation might further explain why fake news spreads faster and further on social media than news that is true (14). An important remark suggests that the mentioned finding could be due to fake news' ability to evoke emotions such as surprise, fear, and disgust (14) that play a crucial role in the cognitive utility of the information (1).

All three investigated factors (truthfulness, cognitive utility, and presentation style) significantly interacted in the process of veracity rating [*F*_(0.83,165)_ = 38.88, *p* < 0.001]. The news items that received the highest veracity scores were true, congruent with the participant's attitude, and presented in a sober manner (Figure 3E). Those news items that received the lowest veracity rating were false, incongruent with the participant's attitude, and presented in a populistic manner (Figure 3E).

In terms of engagement with the news, the most engaging items were congruent with the rater's attitude and presented in a sober manner [*F*_(0.82,163)_ = 27.59, *p* < 0.001]. For such items, we found very little evidence for actual truthfulness to influence the engagement scores (*p* > 0.999). At the same time, those items that were the least engaging were false, incongruent with the rater's attitude, and presented in a populistic way (Figure 3F). Interestingly, the news that was true and sober but incongruent with the rater's attitude was less engaging than the news that was false and soberly presented but congruent (*p* = 0.032, Figure 3F). The results of all remaining *post-hoc* comparisons are presented in Table 1 (for veracity ratings) and Table 2 (for engagement ratings).

**Table 1:** *Post-hoc* tests for three-way ANOVA on factors influencing veracity judgment of news.

**Šídák's multiple comparisons test**	**Mean diff**.	***t***	**Adjusted *P*-value**
Congruent | True | Sober vs. Congruent | True | Populistic	0.36	10.28	<0.001
Congruent | True | Sober vs. Congruent | Fake | Sober	0.71	13.99	<0.001
Congruent | True | Sober vs. Congruent | Fake | Populistic	1.14	18.14	<0.001
Congruent | True | Sober vs. Incongruent | True | Sober	0.19	4.26	<0.001
Congruent | True | Sober vs. Incongruent | True | Populistic	0.87	14.74	<0.001
Congruent | True | Sober vs. Incongruent | Fake | Sober	1.17	19.13	<0.001
Congruent | True | Sober vs. Incongruent | Fake | Populistic	1.58	23.59	<0.001
Congruent | True | Populistic vs. Congruent | Fake | Sober	0.36	6.78	<0.001
Congruent | True | Populistic vs. Congruent | Fake | Populistic	0.79	13.86	<0.001
Congruent | True | Populistic vs. Incongruent | True | Sober	−0.17	3.57	0.012
Congruent | True | Populistic vs. Incongruent | True | Populistic	0.52	10.50	<0.001
Congruent | True | Populistic vs. Incongruent | Fake | Sober	0.81	14.76	<0.001
Congruent | True | Populistic vs. Incongruent | Fake | Populistic	1.23	21.60	<0.001
Congruent | Fake | Sober vs. Congruent | Fake | Populistic	0.43	12.29	<0.001
Congruent | Fake | Sober vs. Incongruent | True | Sober	−0.52	8.58	<0.001
Congruent | Fake | Sober vs. Incongruent | True | Populistic	0.16	2.37	0.408
Congruent | Fake | Sober vs. Incongruent | Fake | Sober	0.46	8.21	<0.001
Congruent | Fake | Sober vs. Incongruent | Fake | Populistic	0.87	14.18	<0.001
Congruent | Fake | Populistic vs. Incongruent | True | Sober	−0.96	13.11	<0.001
Congruent | Fake | Populistic vs. Incongruent | True | Populistic	−0.27	3.88	0.004
Congruent | Fake | Populistic vs. Incongruent | Fake | Sober	0.03	0.42	>0.999
Congruent | Fake | Populistic vs. Incongruent | Fake | Populistic	0.44	7.30	<0.001
Incongruent | True | Sober vs. Incongruent | True | Populistic	0.68	16.06	<0.001
Incongruent | True | Sober vs. Incongruent | Fake | Sober	0.98	20.80	<0.001
Incongruent | True | Sober vs. Incongruent | Fake | Populistic	1.40	24.48	<0.001
Incongruent | True | Populistic vs. Incongruent | Fake | Sober	0.30	7.01	<0.001
Incongruent | True | Populistic vs. Incongruent | Fake | Populistic	0.71	16.87	<0.001
Incongruent | Fake | Sober vs. Incongruent | Fake | Populistic	0.41	12.54	<0.001

DF = 199.

**Table 2:** *Post-hoc* tests for three-way ANOVA on factors influencing engagement with news.

**Šídák's multiple comparisons test**	**Mean diff**.	***t***	**Adjusted *P*-value**
Congruent | True | Sober vs. Congruent | True | Populistic	0.23	6.64	<0.001
Congruent | True | Sober vs. Congruent | Fake | Sober	0.02	0.53	>0.999
Congruent | True | Sober vs. Congruent | Fake | Populistic	0.33	6.38	<0.001
Congruent | True | Sober vs. Incongruent | True | Sober	0.24	5.40	<0.001
Congruent | True | Sober vs. Incongruent | True | Populistic	0.69	11.69	<0.001
Congruent | True | Sober vs. Incongruent | Fake | Sober	0.82	12.09	<0.001
Congruent | True | Sober vs. Incongruent | Fake | Populistic	1.06	14.46	<0.001
Congruent | True | Populistic vs. Congruent | Fake | Sober	−0.20	3.70	0.008
Congruent | True | Populistic vs. Congruent | Fake | Populistic	0.11	2.22	0.540
Congruent | True | Populistic vs. Incongruent | True | Sober	0.01	0.31	>0.999
Congruent | True | Populistic vs. Incongruent | True | Populistic	0.46	10.08	<0.001
Congruent | True | Populistic vs. Incongruent | Fake | Sober	0.60	10.08	<0.001
Congruent | True | Populistic vs. Incongruent | Fake | Populistic	0.83	13.59	<0.001
Congruent | Fake | Sober vs. Congruent | Fake | Populistic	0.31	7.02	<0.001
Congruent | Fake | Sober vs. Incongruent | True | Sober	0.22	3.30	0.032
Congruent | Fake | Sober vs. Incongruent | True | Populistic	0.66	8.95	<0.001
Congruent | Fake | Sober vs. Incongruent | Fake | Sober	0.80	10.51	<0.001
Congruent | Fake | Sober vs. Incongruent | Fake | Populistic	1.04	13.02	<0.001
Congruent | Fake | Populistic vs. Incongruent | True | Sober	−0.09	1.42	0.991
Congruent | Fake | Populistic vs. Incongruent | True | Populistic	0.35	5.31	<0.001
Congruent | Fake | Populistic vs. Incongruent | Fake | Sober	0.49	7.03	<0.001
Congruent | Fake | Populistic vs. Incongruent | Fake | Populistic	0.73	10.71	<0.001
Incongruent | True | Sober vs. Incongruent | True | Populistic	0.45	11.39	<0.001
Incongruent | True | Sober vs. Incongruent | Fake | Sober	0.58	12.05	<0.001
Incongruent | True | Sober vs. Incongruent | Fake | Populistic	0.82	14.36	<0.001
Incongruent | True | Populistic vs. Incongruent | Fake | Sober	0.14	3.50	0.016
Incongruent | True | Populistic vs. Incongruent | Fake | Populistic	0.37	8.80	<0.001
Incongruent | Fake | Sober vs. Incongruent | Fake | Populistic	0.24	7.73	<0.001

DF = 199.

Our results revealed that for the active spreading of information, its consistency with preexisting beliefs is more important than its actual veracity, especially if the information is presented in a sober manner. This observation suggests that one of the most effective ways of spreading misinformation can be based on the Trojan Horse (15) idea. The information designed in this way would have two components: a disinformation carrier and disinformation on its own. The carrier comprises information that is consistent with the opinion of a part of society on one of the most polarizing topics, such as politics or COVID-19, which, through its compliance with the views of the recipients, would be widely shared, dragging with it the actual disinformation on the topic of interest. Thus, the main function of the carriers is to introduce actual disinformation to the discourse on the topic of interest. Indeed, a brief browse of the Internet gives many examples of fake news designed in this way, e.g., that coronavirus has been developed in Ukrainian biolaboratories (16), that Ukrainian president Volodymyr Zelensky is a cousin of Hungarian-born American businessman, and philanthropist supporting progressive and liberal political causes, George Soros (17) or that migrants are spreading new variants of coronavirus (18).

## Limitations

It is essential to report that in the case of several experimental groups, we proceeded with ANOVA, despite the data distribution not being entirely normal, as indicated by the Kolmogorov–Smirnov normality test. Although the normality of the distribution is one of the assumptions of ANOVA, as postulated by Meyers and Well, breaking this assumption should not increase the type I error rate. This is due to the effect of the central limit theorem, which states that the distribution of means and their differences will tend to be normal as sample size increases, even when the distribution of the parent population is not (19, 20).

In studies that heavily rely on *p*-values as a measure of statistical significance, it is important to consider the limitations associated with this approach (21, 22). Relying solely on *p*-values can lead to potential misinterpretations and misuse of statistical results. It is crucial to recognize that *p*-values do not provide a complete picture of the magnitude or practical significance of an effect. Other statistical measures, such as effect sizes and confidence intervals, should be taken into account for a more comprehensive understanding of research findings. The dichotomous interpretation of “significant” and “non-significant” results based solely on *p*-values can oversimplify the complexity of the data. A more nuanced and rigorous approach to statistical analysis is necessary, which involves considering multiple statistical measures and avoiding an exclusive reliance on *p*-values. Given the nature of the current article as a short report, the comprehensive analysis and considerations discussed above regarding the limitations of *p*-values were not extensively applied. Future studies or more in-depth analyses can be undertaken to explore these limitations more extensively.

Finally, it is important to note that susceptibility to misinformation is a highly intricate and interconnected phenomenon that involves both psychocognitive mechanisms and contextual factors about the information. In this study, we attempted to capture some, but certainly not all, of the information features without controlling for any of the psychocognitive mechanisms. As a result, the findings and conclusions should be cautiously generalized, if applicable at all.

## Future directions and conclusion

A number of further avenues of research revolve around these results. First, it would be desirable to confirm these findings using behavioral measures in real-world social media rather than simulations. While it is not ethically acceptable to run experimental studies, by posting false information on social media, it would be possible to do real-world observational work. For example, using a combination of online questionnaires and machine learning methods, one could analyze the past social media sharing behavior of Twitter or Facebook users in the context of the Trojan Horse type of information. For example, some machine learning experts are currently trying to implement psycho-linguistic models in the field of misinformation research (23–25). Another research avenue involves the determination of reasons for knowingly sharing information that is false but congruent with the worldview. Without understanding the cognitive and psychological mechanisms of this behavioral engagement, any interventions aimed at reducing sharing behavior are unlikely to be successful.

The results of this study have practical implications for both researchers and public health institutions. First, it is crucial for researchers to distinguish susceptibility to misinformation based on veracity ratings from behavioral engagement with misinformation. This distinction is vital since the presented results showed that different information factors involve these two types of susceptibility. Additionally, our previous research results (11) pointed to the same importance of susceptibility differentiation from a psychocognitive perspective. Second, as many other studies on fake news concluded, political partisanship is one of the most important factors when deciding about information's truthfulness and willingness to engage with it (26). In this study, we showed that not only political beliefs but also prior beliefs about the COVID-19 pandemic tend to influence susceptibility to misinformation. One of the practical applications of these results might be used during health and vaccination campaigns. Specifically, perhaps instead of debunking maladaptive beliefs, better results could come from an approach that addresses these beliefs with compassion and understanding of individual worldviews.

## Data availability statement

The datasets presented in this study can be found in online repositories. The names of the repository/repositories and accession number(s) can be found below: all data analyzed in this study have been made publicly available via Jagiellonian University Repository, and can be accessed https://doi.org/10.26106/fmdt-fp50.

## Ethics statement

The study was conducted in accordance with all legal requirements regarding the conduct of scientific research of the Kingdom of Norway and the guidelines laid down in the Declaration of Helsinki. Consent was obtained from all subjects. The identical study design was approved by the Bioethics Committee of the Jagiellonian University in Krakow, Poland (1072.6120.66.2021). The patients/participants provided their written informed consent to participate in this study.

## Author contributions

MP designed the news items and the study procedure, conducted the study and statistical analysis, and wrote the draft of the manuscript. KN designed the news items and the study procedure, supervised the statistical analysis, and revised the manuscript. AG designed the news items and the study procedure, conducted the study, and revised the manuscript. JK designed the news items and the study procedure, supervised the statistical analysis, revised the manuscript, and received the funds. MM revised the manuscript and received the funds. JP revised the manuscript and received the funds. RR designed the news items and the study procedure, conducted the statistical analysis, wrote the draft of the manuscript, and received the funds. All authors approved the version to be published and agreed to be accountable for all aspects of the study in ensuring that questions related to the accuracy or integrity of any part of the study are appropriately investigated and resolved.
