# Supplementary material for: Are we willing to share what we believe is true? Factors influencing susceptibility to fake news
Source: Front Psychiatry. 2023 Aug 16;14:1165103. doi: 10.3389/fpsyt.2023.1165103 (PMC10467258; doi:10.3389/fpsyt.2023.1165103)
Supplement: Supplementary file 1 [file Table_1.DOCX]

| **Factorial configuration of the news items** | **Mean** | **SD** | **Min** | **Max** | **Skewness** | **Kurtosis** | **Cronbach's α [95% CI]** |
| --- | --- | --- | --- | --- | --- | --- | --- |
| False \| Populistic \| Congruent | 3,04 | 0,85 | 1,20 | 5,20 | 0,18 | -0,32 | 0,86 [0,82 ; 0,88] |
| False \| Sober \| Congruent | 3,48 | 0,70 | 1,30 | 4,90 | -0,29 | -0,15 | 0,77 [0,72 ; 0,82] |
| False \| Populistic \| Incongruent | 2,60 | 0,84 | 1,00 | 5,10 | 0,43 | -0,15 | 0,83 [0,79 ; 0,86] |
| False \| Sober \| Incongruent | 3,02 | 0,79 | 1,00 | 5,00 | -0,11 | -0,37 | 0,80 [0,75 ; 0,84] |
| True \| Populistic \| Congruent | 3,83 | 0,59 | 1,60 | 5,50 | -0,56 | 1,27 | 0,59 [0,50 ; 0,67] |
| True \| Sober \| Congruent | 4,19 | 0,57 | 1,70 | 5,70 | -0,40 | 1,81 | 0,66 [0,58 ; 0,73] |
| True \| Populistic \| Incongruent | 3,31 | 0,79 | 1,30 | 5,80 | 0,07 | -0,12 | 0,80 [0,76 ; 0,84] |
| True \| Sober \| Incongruent | 4,00 | 0,64 | 1,50 | 5,70 | -0,66 | 1,47 | 0,72 [0,66 ; 0,78] |

**Supplementary Materials**

**Descriptive Statistics of Veracity Ratings**

**Descriptive Statistics of Engagement Ratings**

| **Factorial configuration of the news items** | **Mean** | **SD** | **Min** | **Max** | **Skewness** | **Kurtosis** | **Cronbach's α [95% CI]** |
| --- | --- | --- | --- | --- | --- | --- | --- |
| False \| Populistic \| Congruent | 3,03 | 1,26 | 1,00 | 5,6o | 0,05 | -1,02 | 0,92 [0,90 ; 0,94] |
| False \| Sober \| Congruent | 3,34 | 1,27 | 1,00 | 6,00 | -0,12 | -0,69 | 0,93 [0,91 ; 0,94] |
| False \| Populistic \| Incongruent | 2,30 | 1,15 | 1,00 | 5,10 | 0,65 | -0,50 | 0,93 [0,91 ; 0,94] |
| False \| Sober \| Incongruent | 2,53 | 1,16 | 1,00 | 5,15 | 0,45 | -0,76 | 0,93 [0,91 ; 0,94] |
| True \| Populistic \| Congruent | 3,13 | 1,24 | 1,00 | 5,50 | -0,06 | -0,99 | 0,91 [0,89 ; 0,93] |
| True \| Sober \| Congruent | 3,36 | 1,24 | 1,00 | 5,95 | -0,24 | -0,77 | 0,93 [0,91 ; 0,94] |
| True \| Populistic \| Incongruent | 2,67 | 1,21 | 1,00 | 5,20 | 0,38 | -0,94 | 0,93 [0,92 ; 0,95] |
| True \| Sober \| Incongruent | 3,12 | 1,24 | 1,00 | 5,75 | -0,01 | -0,84 | 0,93 [0,91 ; 0,94] |
